# Neonatal McCune‐Albright Syndrome: A Unique Syndromic Profile With an Unfavorable Outcome

**DOI:** 10.1002/jbm4.10134

**Published:** 2019-01-15

**Authors:** Alessandro Corsi, Natasha Cherman, David L Donaldson, Pamela G Robey, Michael T Collins, Mara Riminucci

**Affiliations:** ^1^ Department of Molecular Medicine Sapienza University Rome Italy; ^2^ Skeletal Biology Section National Institute of Dental and Craniofacial Research National Institutes of Health Bethesda MD USA; ^3^ Department of Pediatrics University of Utah, School of Medicine Salt Lake City UT USA; ^4^ Skeletal Disorders and Mineral Homeostasis Section National Institute of Dental and Craniofacial Research National Institutes of Health Bethesda MD USA

**Keywords:** MCCUNE‐ALBRIGHT SYNDROME, GNAS, CUSHING SYNDROME, CHOLESTASIS, NEONATAL

## Abstract

Somatic gain‐of‐function mutations of *GNAS* cause a spectrum of clinical phenotypes, ranging from McCune‐Albright syndrome (MAS) to isolated disease of bone, endocrine glands, and more rarely, other organs. In MAS, a syndrome classically characterized by polyostotic fibrous dysplasia (FD), café‐au‐lait (CAL) skin spots, and precocious puberty, the heterogenity of organ involvement, age of onset, and clinical severity of the disease are thought to reflect the variable size and the random distribution of the mutated cell clone arising from the postzygotic mutation. We report a case of neonatal MAS with hypercortisolism and cholestatic hepatobiliary dysfunction in which bone changes indirectly emanating from the disease genotype, and distinct from FD, led to a fatal outcome. Pulmonary embolism of marrow and bone fragments secondary to rib fractures was the immediate cause of death. Ribs, and all other skeletal segments, were free of changes of typical FD and fractures appeared to be the result of a mild‐to‐moderate degree of osteopenia. The mutated allele was abundant in the adrenal glands and liver, but not in skin, muscle, and fractured ribs, where it could only be demonstrated using a much more sensitive PNA hybridization probe‐based FRET (Förster resonance energy transfer) technique. Histologically, bilateral adrenal hyperplasia and cholestatic disease matched the abundant disease genotype in the adrenals and liver. Based on this case and other sporadic reports, it appears that gain‐of‐function mutations of *GNAS* underlie a unique syndromic profile in neonates characterized by CAL skin spots, hypercortisolism, hyperthyroidism, hepatic and cardiac dysfunction, and an absence (or latency) of FD, often with a lethal outcome. Taken together, our and previous cases highlight the phenotypic severity and the diagnostic and therapeutic challenges of MAS in neonates. Furthermore, our case specifically points out how secondary bone changes, unrelated to the direct impact of the mutation, may contribute to the unfavorable outcome of very early‐onset MAS. © 2018 The Authors *JBMR Plus* published by Wiley Periodicals, Inc. on behalf of American Society for Bone and Mineral Research.

## Introduction

Somatic, missense gain‐of‐function mutations of the *GNAS* gene, encoding the α‐subunit of the stimulatory G protein, Gαs, are known to cause a varied spectrum of clinical expressions, including the McCune‐Albright syndrome (MAS), isolated monostotic or polyostotic fibrous dysplasia (FD) of bone, isolated endocrine tumors, the syndromic association of muscular myxomas with FD, and isolated myxomas.^1^, ^2^, ^3^, ^4^, ^5^, ^6^, ^7^, ^8^, ^9^, ^10^, ^11^, ^12^ Recently, the same mutations have also been reported in a spectrum of low‐grade and benign hepato‐biliary, pancreatic, and gastrointestinal neoplasias occuring either with or without other clinical features of MAS.^11^, ^12^, ^13^, ^14^ In all the different clinical contexts, the *GNAS* mutation leads to activation and inappropriate activity of cells and tissues normally stimulated through receptors that are coupled to the Gα‐cAMP‐protein‐kinase A dependent pathway.^1^, ^2^

In MAS, affected tissues and cell types include derivatives of the different germ layers.^1^, ^8^ Even though polyostotic FD, café‐au‐lait skin spots, and hyperfunctional endocrinopathies (in particular, peripheral precocious puberty) represent the typical clinical features of the disease, other tissues and organs can be affected.^8^, ^9^, ^10^, ^11^, ^12^, ^15^ The variability in the pattern of tissue and organ involvement, the age of onset and the severity of the clinical disease are generally thought to reflect the variable size and the random distribution to diverse tissues/organs of the clone of mutated embryonic cells arising from the postzygotic mutation.^3^, ^8^, ^16^ However, other factors, such as (1) the level of expression of the mutant allele in different tissues^17^, (2) the activity of counterregulatory molecular mechanisms such as relevant tissue‐specific phosphodiesterases^1^, ^18^, (3) the cell‐specific effects of the increased cAMP production^15^, (4) the differential survival of mutated cells across the body and along development^8^ and (5) epigenetic determinants such as tissue‐specific imprinting of the *GNAS* gene,^19^, ^20^, ^21^ may all represent important additional determinants of the different disease burden of MAS patients.

Presentation of MAS in neonatal life is a rare and often lethal event.^3^, ^22^, ^23^, ^24^, ^25^, ^26^, ^27^, ^28^, ^29^, ^30^, ^31^, ^32^, ^33^, ^34^ We report here a case of multiorgan disease caused by a gain‐of‐function mutation of *GNAS* that presented at birth and had an unfavorable outcome. Major pathological changes were observed in the adrenal glands and liver, consistent with the detection of the greatest concentration of the disease genotype at these sites. No typical features of FD were detected. Nonetheless, an osteopenic bone phenotype, most likely secondary to hypercortisolism, was the major determinant of lethality, with multiple pulmonary marrow emboli originating from fractures of osteopenic ribs. This case and previous reports of MAS with very early onset^3^, ^22^, ^23^, ^24^, ^25^, ^26^, ^27^, ^28^, ^29^, ^30^, ^31^, ^32^, ^33^, ^34^ highlight a syndromic profile of the disease that appears to be unique to the neonatal period, and requires a prompt diagnosis, but may be difficult to recognize. In addition, our case highlights the importance of bone changes that, in the absence of FD or any other direct impact of the disease genotype on bone, may contribute to the lethal outcome of MAS in neonatal patients.

## Case Presentation

**Figure 1:**
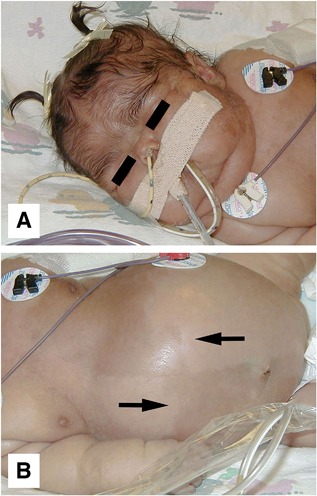
(*A*) The patient presented at birth with a cushingoid facies and hirsutism. (*B*) A large abdominal macular area of hyperpigmentation respecting the midline was also observed (arrows).

**Figure 2:**
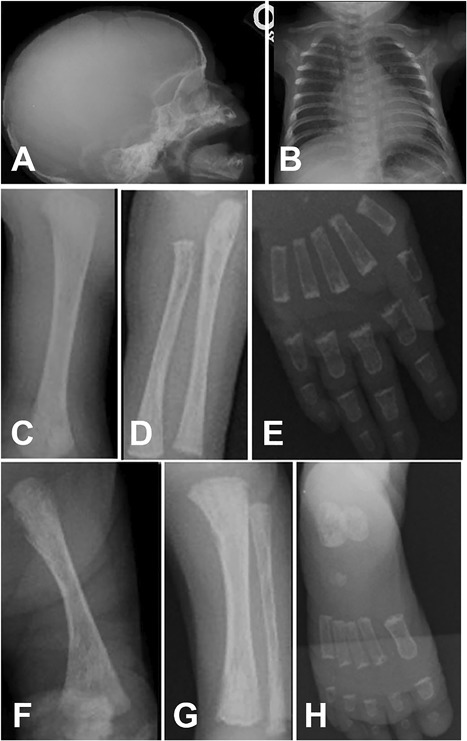
The radiographic survey on admission failed to show fibrous dysplasia lesions. In contrast, a mild‐to‐moderate degree of osteopenia was observed in diverse skeletal segments. A = cranium; B = chest; C = humerus; D = radius and ulna; E = carpal and metacarpal bones; F = femur; G = tibia and fibula; H = tarsal and metatarsal bones.

The female child was the product of a nonconsanguineous union, born at 40 weeks of gestation after an uneventful pregnancy. The mother was 32 years old (gravidity 3 and parity 3) with three living, healthy children. At birth, meconium aspiration was noted along with difficulty in maintaining oxygenation. Apgar scores were 6 at 1 min and 8 at 5 min. She was born small for her gestational age (length 43 cm, weight 1715 g, head circumference 28 cm; all below the third centile). The child began to experience respiratory distress and 3 hours after birth was admitted to the neonatal critical care unit. On admission, the child was tachycardic, tachypneic, and icteric. She had a cushingoid appearance, hypertrichosis, and large macular areas of skin hyperpigmentation with a “coast of Maine” profile on the proximal extremities and on the abdomen, where they tended to respect the midline (Fig. 1). A III/VI systolic murmur was noted, and the liver edge was palpable 4 cm below the right costal margin. Based on the initial concern of sepsis, the patient was treated empirically for bacterial and viral infections with intravenous ampicillin, gentamycin, and acyclovir. The pattern of the pigmented areas raised the suspicion of MAS, which was confirmed when testing documented hyperthyroidism and hypercortisolism (Table 1). Other abnormalities included hepatobiliary dysfunction (increase in conjugated bilirubinemia, hypertransaminasemia), anemia, hyperglycemia, and hypertension. Radiographic studies of the skeleton showed a mild‐to‐moderate degree of osteopenia, but did not reveal findings consistent with FD (Fig. 2). An echocardiogram demonstrated abnormal moderate‐to‐severe tricuspid regurgitation, right ventricular enlargement and hypertrophy, a small atrial septal defect with right‐to‐left low velocity shunting, and a moderately sized patent ductus arteriosus with low‐velocity left‐to‐right shunting. A 24‐hour Holter electrocardiogram showed only persistent sinus tachycardia at a constant rate of approximately 158 bpm, even during sleep. Adrenalectomy was planned subject to improvement of general condition, and treatment with methimazole was effective in controlling the hyperthyroidism. With the normalization of the thyroid hormone levels, there was a concomitant resolution of the tachycardia. An attempt was made to decrease the adrenal cortisol production by blocking adrenal steroid synthesis with ketoconazole (1.0 mg twice a day). This was abandoned when serum γ‐glutamyltransferase (GGT) levels rose from 93 to 324 U/L (normal 0 to 242 U/L), at a time when other measures of liver toxicity were falling. With the discontinuation of ketoconazole, GGT normalized. The child was discharged on day 29, but over the next 3 months, several brief hospitalizations were necessary for failure to thrive, poor fluid and nutrition intake, and respiratory infections. Serum cortisol values continued to rise, reaching a peak of 100 μg/dL and she developed respiratory distress as the result of *Pneumocystis carinii* pneumonia. She was hospitalized, intubated, and placed on mechanical ventilation. Blockade of adrenal steroid production was attempted with metyrapone, 300 mg/m^2^/day given three times daily, increasing the dose to 600 mg/m^2^/day. With blockage, the morning cortisol level was maintained between 16.8 to 39.3 μg/dL (normal 5 to 25 μg/dL). Treatment with trimethoprim/sulphamethoxazole led to the apparent resolution of the *Pneumocystis carinii* pneumonia, as confirmed by clearing of chest X‐ray and sputum. Despite this, the numerous attempts to wean her from mechanical ventilation were unsuccessful and she died. A postmortem examination was performed. The most relevant gross findings were the enlargment of the adrenal glands, an enlarged green‐colored liver, and rib fractures.

**Table 1:** Early Main Postpartum Laboratory Test Results

Test	Value	Normal range
TSH	<0.05 mIU/mL	0.32–5.00
Free T4	2.67 ng/dL	0.71–1.85
AM cortisol	71.1 μg/dL	5.0–25.0
AM cortisol after 0.5 mg dexamethasone at MN	55.1 μg/dL	<5
AM cortisol after 2.0 mg dexamethasone at MN	52.5 μg/dL	<5
ACTH (random value)	19 pg/mL	9–54
Total bilirubin	14.2 mg/dL	0.1–1.0
Conjugated bilirubin	10.4 mg/dL	0.0–0.3
ALT	1422 U/L	5–45
AST	618 U/L	20–60
Alkaline phosphatase	340 U/L	62–368
Ionized calcium	1.23 mmol/L	1.20–1.48
Magnesium	2.71 mg/dL	1.5–2.2
Phosphorus	3.5 mg/dL	3.5–6.5
Hematocrit	32.6%	42.0–56.0

TSH = thyroid stimulating hormone; T4 = thyroxine; AM = morning; MN = midnight; ACTH = adrenocorticotropin hormone; ALT = alanine transaminase; AST = aspartate transaminase.

## Material and Methods

### Tissue samples

The mother gave consent for an autopsy and the study of specimens under a research protocol approved by the National Institute of Dental and Craniofacial Research Institutional Research Board (98‐D‐0145). At the end of the autopsy, samples from different organs were immediately frozen in liquid nitrogen for mutation analysis or immediately fixed in 4% formaldehyde (freshly made from paraformaldehyde) in 0.1 mol/L phosphate buffer (pH 7.2) for histology and immunohistochemistry.

### Mutation analysis

Mutation analysis was performed on samples from bone (fractured rib), skin, skeletal muscle, adrenal glands, liver, kidney, and ovary. After extraction of genomic DNA from the frozen samples (Qiagen DNeasy Tissue Kit, Qiagen Inc, Valencia, CA, USA), a target sequence of the Gαs gene including the R201 codon was amplified using both standard PCR and the polypeptide nucleic acid (PNA)‐based PCR method previously described.^5^, ^35^, ^36^

The standard PCR reaction was performed using the following primers: 5’‐TGACTATGTGCCGAGCGA (sense) and 5’‐AACCATGATCTCTGTTATATAA (antisense), (GenBank Accession no. AH002748, bases 3258–3275 and 3507–3528, respectively), which amplify a 270‐bp fragment of the Gαs gene. After a denaturation step of 15 min at 95°C, the target sequence was amplified for 35 cycles at the following temperatures: 95°C for 30 s, 55°C for 30 s, and 72° for 30 s, followed by 7 min of final extension at 72°C. A fragment of 326 bp was chosen as the target for the PNA‐based PCR and amplified using the following primers: 5’‐GTTTCAGGACCTGCTTCGC (sense) and 5’‐GCAAAGCCAAGAGCGTGAG (antisense) (GenBank Accession no. AH002748, bases 3406–3424 and 3714–3732, respectively). A PNA sequence complementary to the normal allele (amino‐terminal 5’‐CGCTGCCGTGTC carboxy‐terminal 3’) was added to the reaction mixture (2 μg) to block the amplification of the normal allele and allow the selective amplification of the mutated allele. After 15 min of denaturation at 94°C, 40 cycles of amplification were performed at the following: 94°C for 30 s, 68°C for 60 s (to allow the binding of the PNA), 55°C for 30 s (to allow the binding of the sense primer), and 72°C for 60 s. The final extension was 7 min at 72°C.

In both cases, the PCR product was purified (Wizard PCR Preps DNA Purification System; Promega, Madison, WI, USA) and then sequenced using rhodamine dye‐terminator cycle sequencing with Ampli Taq and the Perkin Elmer Applied Biosystem 377 automated sequencer (Perkin Elmer, Norwalk, CT, USA).

### Histology and immunohistochemistry

Tissue samples for histology were routinely processed for paraffin embedding. Bone samples were processed after decalcification as described previously.^37^ From each paraffin block, 3‐ to 5‐µm sections were prepared and stained with hematoxylin and eosin or used for immunohistochemistry.

Immunohistochemical stains were performed on sections obtained from the adrenal glands, liver, and pancreas. Different primary antibodies were used: (1) rabbit anti‐human 3β‐hydroxysteroid dehydrogenase (3β‐HSD; kindly provided by Prof. JL Thomas, Washington University, St. Louis, MO, USA); (2) rabbit anti‐human Gαs (LF‐129; kindly provided by Dr. LW Fisher, National Institute of Dental and Craniofacial Research, National Institutes of Health, Bethesda, MD, USA); (3) mouse anti‐cytokeratin 7 (CK7, Clone OV‐TL 12/30; Dako, Glostrup, Denmark); and (4) mouse anti‐chromograninA (CgrA, Clone NCL‐Crom‐430; Novocastra, Newcastle, UK). Sections were deparaffinized and exposed for 30 min to 0.3% H_2_O_2_ to inhibit endogenous peroxidase. Sections to be stained with LF‐129 (1:200 in PBS + 1% BSA) and anti‐3β‐HSD antisera (1:20 in PBS + 1% BSA) were incubated for 30 min at room temperature with undiluted normal swine serum followed by incubation with the primary antibody for 2 hours at room temperature. Sections to be stained with anti‐CK7 (1:50 in PBS + 1% BSA) and anti‐CgrA (1:100 in PBS + 1% BSA) antisera were exposed twice for 5 min at maximum power in a 750W microwave oven, incubated at room temperature for 30 min with undiluted normal rabbit serum, followed by 2‐hour incubation with the primary antibody. After repeated washing with PBS, sections were incubated for 30 min with biotin‐conjugated swine anti‐rabbit IgG (1:200 in PBS + 1% BSA) or rabbit anti‐mouse IgG (1:200 in PBS + 1% BSA) and then exposed for 30 min to peroxidase‐conjugated extravidin (1:50 in PBS + 1% BSA). The peroxidase reaction was developed using 3,3'‐diaminobenzidine tetrahydrochloride (Sigma, St. Louis, MO, USA) as substrate.

## Results

### Analysis of *GNAS* mutation and mutational load in different tissues and organs

**Figure 3:**
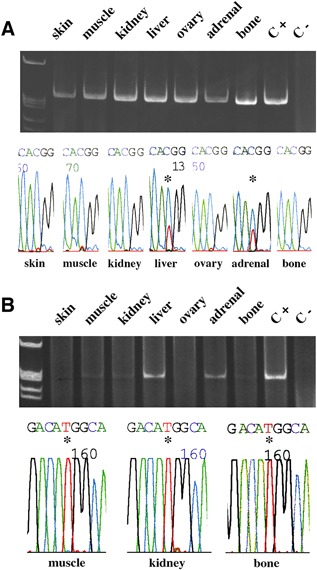
(*A*) Standard PCR (upper panel) and sequence of the relevant region of the amplicon (bottom panel). The transition G > A (C > T in the antisense sequence; asterisks) indicative of the R201H mutation was detected in the liver and adrenal samples, but not in skin, skeletal muscle, kidney, ovary, and bone. (*B*) PNA‐based PCR (upper panel) and sequence of the relevant region of the amplicon (bottom panel). The presence of a low amount of amplification product in skeletal muscle, kidney, and bone (fractured rib) revealed the presence of the mutated sequence, which was confirmed by DNA sequencing (asterisks).

Mutation analysis was conducted on gDNA extracted from skin, skeletal muscle, kidney, liver, ovary, adrenal glands, and bone. We first performed a standard PCR to amplify a 270‐bp target sequence including the CGT codon in exon 8 of *GNAS* (R201) followed by direct and reverse sequencing of the amplicon. By using this approach, a G > A (R201H) mutation was detected in the adrenal glands and liver, but not in the skin, skeletal muscle, kidney, ovary, and bone (Fig. 3 *A*). However, it is known that because of the variability in the degree of mosaicism in different tissues and organs,^1^, ^2^, ^8^, ^9^, ^10^, ^11^, ^12^ the sensitivity of the standard PCR approach (25% to 50% mutant cells) may not be sufficient to detect the disease genotype at sites in which a relatively low number of mutated cells is present. Therefore, to better assess the presence and distribution of the mutation and to obtain information on the relative load of mutated cells in different tissues, we also performed a PCR analysis with PNA clamping to inhibit the amplification of the wild‐type allele. By using this approach, which may demonstrate an estimated load of about 1 mutated cell out of 200,^5^, ^35^ followed by sequencing of the amplification product, mutated cells were also detected in skeletal muscle, kidney, and bone (Fig. 3 *B*).

### Direct and indirect effects of GNAS R201H on different tissues and organs

**Figure 4:**
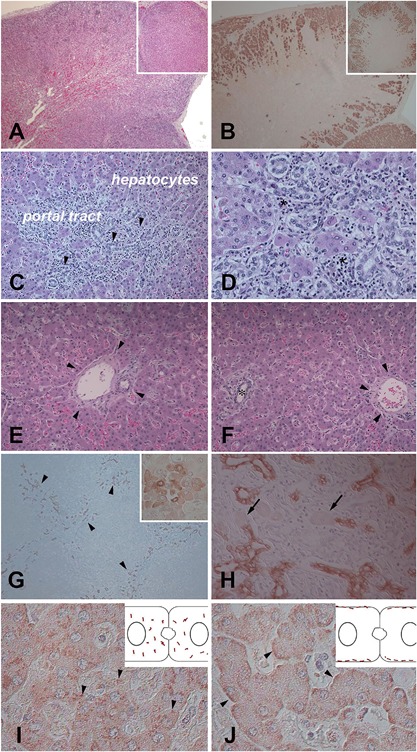
(*A,B*) Pathological changes in the adrenal glands. The glands were hyperplastic (*A*) and the lesional cells showed intense and extensive immunoractivity for 3β‐HSD, consistent with their steroidogenic competence (*B*). The inserts illustrate serial sections of an adrenal hyperplastic nodule. (C–J) Pathological changes in the liver. Some portal tracts showed a prominent ductular reaction (*C*; arrowheads) associated with an inflammatory infiltrate enriched in polymorphonuclear leukocytes (*D*; asterisks). Other portal tracts were devoid of biliary ducts (*E*; the arrowheads indicate the boundary of the portal tract**)** and intralobular ectopic biliary ducts were also detected (*F*; the asterisk identifies the lumen of the biliary duct and the arrowheads indicate the boundary of the nearest portal tract). Immunostaining for CK7 highlighted the ductular reaction (*G* **;** arrowheads), revealed biliary metaplasia of hepatocytes (insert in *G*) and demonstrated that the newly generated ductules were lined by both CK7 positive cholangiocytes and CK7 negative hepatocytes (*H*; arrows indicate CK7 negative hepatocytes). Immunostaining for Gαs demonstrated a rough granular pattern of distibution of the protein within the cytoplasm of the patient's hepatocytes (*I*; arrowheads). In contrast, the cytoplasmic distribution of the protein in a control liver section was predominantly along the sinusoidal domain of the hepatocytes (*J*; arrowheads). The inserts in *I* and *J* show by drawings the distribution of the protein in the cytoplasm of two adjacent hepatocytes.

In agreement with the distribution of the disease genotype and the mutational load observed in the different organs, histological analysis revealed major pathological changes in the adrenal glands and in the liver. Consistent with the clinical features of hypercortisolism and with previous reports of neonatal MAS,^3^, ^22^, ^23^, ^24^, ^25^, ^27^, ^28^, ^29^, ^30^, ^32^, ^33^ adrenal glands featured bilateral cortical hyperplasia (Fig. 4 *A*) in which occasional nodules were also noted. Immunostaining for 3β‐HSD was intense and extensive (Fig. 4 *B*), thus indicating the steroidogenic competence of the adrenal cells.^38^, ^39^ In the liver, pathological changes were consistent with a cholestatic disease and characterized by altered number and distribution of the biliary ducts. The majority of the portal tracts was expanded and characterized by a prominent ductular reaction associated with an inflammatory infiltrate mainly consisting of polymorphonuclear leucocytes (Fig. 4 *C*, *D*). Conversely, the biliary ducts were absent in some portal tracts (Fig. 4 *E*). Finally, bile ducts were frequently observed far from the portal tracts within the hepatic lobule (Fig. 4 *F*). In agreement with this finding, bile plugs within the biliary canaliculi and feathery degeneration of hepatocytes were also noted. Immunostaining for CK7 (1) better highlighted the ductular reaction (Fig. 4 *G*), (2) revealed hepatocytes undergoing biliary metaplasia (insert in Fig. 4 *G*) and (3) demonstrated that the newly generated ducts were lined by both CK7 positive cholangiocytes and by CK7 negative hepatocytes (Fig. 4 *H*). No giant cell transformation of hepatocytes was observed. Immunostaining for Gαs demonstrated a granular pattern of distribution of the protein in the cytoplasm of the hepatocytes in contrast with the predominant distribution along the sinusoidal domain of the hepatocytes in age‐ and gender‐matched control (Fig. 4 *I*, *J*).

**Figure 5:**
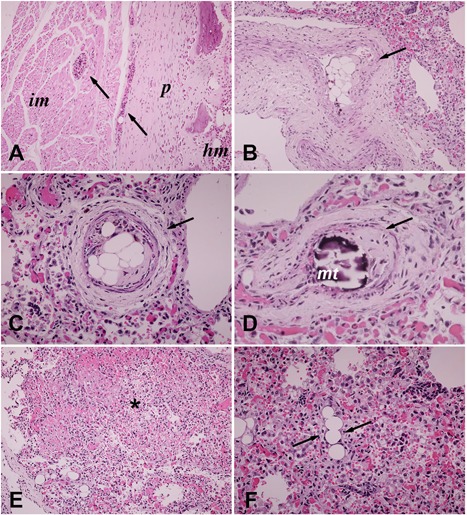
(A) Marrow emboli (arrows) within veins of the rib periosteum (*p*) and of intercostal muscle (*im*). Please note the thinned discontinuous bony cortex that encased a nonfibrotic, hematopoietic marrow cavity *(hm*). (*B–D*) Intraparenchymal branches of pulmonary arteries (arrows) with marrow emboli are shown in *B* and *C* and an embolus with fragments of mineralized tissue (*mt*), consistent with bone, is shown in *D*. (*E*) Small area of lung infarction (asterisk). (*F*) Fatty embolism in intraparenchymal lung capillaries (arrows).

Consistent with the antemortem radiographic findings, no histological evidence of FD was observed in the ribs, where a thinned and discontinuous bony cortex encased a nonfibrotic, hematopoietic marrow cavity. Of note, multiple marrow emboli were detected in the veins of the rib periosteum and intercostal muscles (Fig. 5 *A*), whereas in the lungs, multiple marrow emboli and fragments of mineralized tissue, consistent with bone, were found impacted in branches of pulmonary arteries (Fig. 5 *B–D*). In addition, small areas of lung infarction (Fig. 5 *E*) and fatty embolism in lung capillaries (Fig. 5 *F*) were noted. Taken together, these findings were consistent with the multiple emboli that originated from fractured ribs and reached the pulmonary circulation, providing an explanation for the terminal dependence on mechanical ventilation in the face of resolved *Pneumocystis carinii* pneumonia and a plausible cause of death. They also indicated that the rib fractures that were not apparent in the skeletal X‐ray survey on admission, but were observed at autopsy, were not dependent on the presence of FD, but rather were likely caused by glucocorticoid‐related skeletal disease.

**Figure 6:**
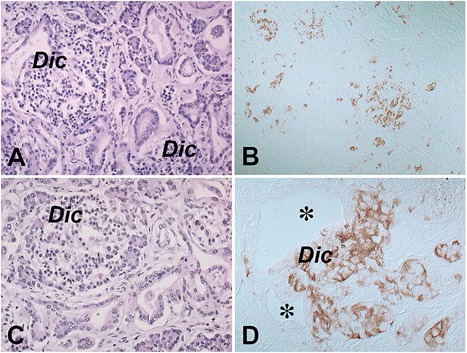
(*A,C*) Ductulo‐insular complexes in the pancreas as shown by hematoxylin and eosin staining. (*B,D*) Immunostaining for CgrA highlights the increased and haphazardly distributed islet cell clusters (*B*) and the budding of CgrA‐positive islet cells from the epithelium of pancreatic ducts (*D*; asterisks) with formation of ductulo‐insular complexes. Dic = ductulo‐insular complex.

Additional relevant pathological changes provided by histological analysis included nephrocalcinosis, and in the pancreas (Fig. 6 *A–D*), the abundance of ductulo‐glandular complexes (ie, diffuse nesidioblastosis, congenital islet hyperplasia) that were highlighted by CgrA immunostaining (Fig. 6 *B*, *D*).

## Discussion

We report here a case of neonatal lethal multiorgan disease caused by a gain‐of‐function mutation (R201H) in the *GNAS* gene. The patient presented at birth with large café‐au‐lait (CAL) skin macules, hypercortisolism, hyperthyroidism, and liver and cardiac dysfunction. Medical control of the hyperthyroidism was pharmacologically obtained, but before successful adrenal blockade could be achieved, the child died from respiratory distress. Even though FD lesions were not detected, a bone phenotype consisting in a mild‐to‐moderate degree of osteopenia was the major determinant of lethality caused by pulmonary marrow and bone fragment embolisms originating from fractures of the fragile ribs.

Neonatal presentation of MAS is rare and few cases have been reported^3^, ^22^, ^23^, ^24^, ^25^, ^26^, ^27^, ^28^, ^29^, ^30^, ^31^, ^32^, ^33^, ^34^ since the first descriptions by McCune and Bruch^40^ and by Abright and colleagues.^41^ In this context (Table 2), CAL skin macules, Cushing syndrome (CS), hyperthyroidism, and liver and cardiac dysfunction are the most common clinical manifestations. In contrast, precocious puberty and FD, two of the three “classic” features of MAS, are usually absent. Importantly, the outcome of MAS presenting in the neonatal age is often unfavorable, leading to early death based on a combination of unusual nonendocrine and endocrine clinical manifestions. A high index of suspicion, prompt diagnosis, and early interventation can be life‐saving. Clinicians should be alerted by the evidence of CAL skin macules that are frequently present at birth and may be the clue for the recognition of the syndrome,^3^, ^9^, ^10^, ^22^, ^25^, ^28^, ^30^, ^33^ in particular, if associated with the early appearance of cholestatic hepatobiliary dysfunction^26^, ^31^, ^42^ and/or endocrine hyperfunctions that are rare at the neonatal age, such as CS and hyperthyroidism.^43^, ^44^, ^45^ In the case reported here, the presence and the characteristic pattern of the skin hyperpigmented areas raised the suspicion of MAS, which was confirmed when testing documented hyperthyroidism and hypercortisolism. Adrenal hyperfunction and hepatobiliary dysfunction were the main clinical expressions of the disease. In addition, the adrenal glands and liver harbored higher numbers of mutated cells compared to the other tissues/organs that were analyzed.

**Table 2:** Clinical Synopsis of Reported Cases of Neonatal McCune‐Albright Syndrome

Ref. #	Gender/age	CS	HBD	HT	HD	CAL	PP	Skeletal system	Outcome
3 (case 1)	M/2 days	+ (BA at 2 years)	+	+	+	+ (Birth)	–	Biopsy‐proven FD at 2 months	Died at 3 years and 10 months (unexplained cardiac arrest, cause of death not revealed at autopsy)
3 (case 2)	F/10 days	+ (BA at 3 months)	+	+	+	+ (2 Months)	+ (<1 Year)	Severe osteopenia at 2 months; severe osteopenia, rickets, and progression of severe PFD with leg fracture at 6 months	Died at 2 years (sudden death)
22	F/birth	+ (BA at 8 months)	–	–	–	+ (Birth)	+ (5 Months)	Generalized demineralization with coarse trabeculation of metaphyses, stippling of the epiphyses, and irregular luciences in both ilia and ischia at 6 months	NA
23	M/7 days	+ (BA at 6 weeks)	NA	+	NA	NA	NA	PFD in infancy	Lost to follow‐up at 5.5 months
24	F/1 month	+	+	+	+	+ (1 Months)	–	Widening of the metaphyses and polyostotic irregular lucencies at 1 months	Died at 132 days (cardiac failure)
25 (case 4)	M/birth	+ (BA at 5 months)	–	–	+	+ (Birth)	–	Cystic changes in the femura consistent with PFD at 4 months	Died at 8 months (sudden death)
26 (case 1)	M/2 days	–	+	–	–	+ (4 Months)	–	PFD at 2.5 years	Alive at 11 years, without fractures and endocrinological dysfunction
26 (case 2)	F/4 days	–	+	–	–	+ (4 Years)	–	PFD with fracture of the right femur neck at 4 years	Alive at 9 years with recurrent fractures of the right femur neck, but no endocrinological dysfunction
27 (case 9)	NA/birth	+ (BA, age NA)	+	+	+	+ (Age NA)	–	PFD (age NA)	Died at 14 months (sepsis)
28 (case 1)	F/birth	+ (BA at 7 months)	+	+	+	+ (Birth)	–	Neonatal PFD	Died at 9 months (unspecified complications of MAS and CS)
28 (case 5)	F/2 months (in utero in retrospect)	+ (BA at 3 months)	+	+	+	+ (2 Months)	+ (7 Months)	PFD at 18 months	Died at 2 years (sudden death)
29	F/1 month	+ (BA at 3 months)	+	+	+	+ (2 Months)	+ (4 Months)	PFD at 2 months	Died at 15 months (sudden death, probable adrenal crisis)
30	F/17 days	+	+	+	+	+ (Birth)	–	Generalized reduction of bone density, irregular ossification with areas of radiolucence surrounded by sclerosis, most evident in radius and ulna	Died at 4 months (acute respiratory failure)
31	F/28 days	–	+	–	–	+ (28 Days)	–	Extensive, unusual lesion on the right zygoma, predisposing FD at 28 days	NA
32	F/18 days	+ (BA at 4 months)	+	+	+	+ (Between 5 and 26 Months)	+ (22 Months)	Diffuse and severe skeletal dysplasia then (between 5 and 26 months) femur fractures caused by severe osteoporosis from hypercortisolism and phosphate wasting, though radiographic imaging alone was unable to exclude FD	Alive at 29 months, developmental cognitive and motor delays
33	F/birth	+ (BA, age NA)	+	+	+	+ (Birth)	+ (6 Months)	PFD and severe osteopenia with multiple fractures at 6 months	Died at 8 months (multiorgan failure)
34	F/birth	–	+	–	+	+ (14 Months)	+ (14 Months)	Spontaneous femur fracture between 1 and 10 months and PFD at 21 months	Alive at 27 months (liver transplantation at 10 months and recurrent femur fractures)
Present case	F/birth	+	+	+	+	+ (Birth)	–	Mild‐to‐moderate osteopenia, rib fractures	Died at 4 months (lung embolism of bone marrow and mineralized fragments consistent with bone)

CS = Cushing syndrome/hypercortisolism; HBD = hepatobiliary dysfunction; HT = hyperthyroidism; HD = heart dysfunction; CAL = café‐au‐lait macules; PP = precocious puberty; BA = bilateral adrenalectomy; FD = fibrous dysplasia; PFD = polyostotic fibrous dysplasia; NA = not available; MAS = McCune‐Albright syndrome.

CS is the rarest of endocrine abnormalities found in MAS with an estimated prevalence of 7.1%.^27^ It virtually always occurs in very young patients.^9^, ^10^, ^27^ The reported median age at diagnosis is 3.1 months.^27^ Because of the potential devastating course of CS at the neonatal age, prompt recognition and appropriate care are essential.^44^, ^46^ In the context of neonatal MAS, rapid assessment of significant comorbidities must be performed, including hyperthyroidism and, in particular, cardiac and hepatobiliary dysfunction that are negative prognostic markers and may suggest the need for immediate adrenalectomy.^27^ Evaluation of complications of cortisol excess including hypertension, hyperglycemia, and nephrocalcinosis, and prophylaxis for opportunistic infections (first, *Pneumocystis carinii*) must be conducted as well.^9^, ^10^, ^27^ CS is unique among MAS‐related endocrinopathies because of its tendency to resolve spontaneously,^27^, ^47^ most likely because of the physiological involution of the adrenal fetal zone in the first year of life.^28^, ^48^ Although in our case we could not establish conclusively whether the adrenal hyperfunction was corticotropin dependent or independent, the hypercortisolism was central to a very unusual pathway of disease lethality caused by the development of rib fractures. In fact, the absence of clinicoradiographic and histological evidence of focal lesions dispelled the possibility of FD as the cause of the fractures. Thus, it could be concluded that the excess of cortisol, likely with the association of other factors including cholestasis^49^ and hypoxia,^50^ led to a mild‐to‐moderate degree of osteopenia that contributed significantly to the development of the rib breakage, possibly during assisted ventilation. This in turn caused multiple embolizations of marrow and mineralized tissue fragments in the pulmonary circulation as a terminal event documented by postmortem findings. Even though it cannot be proved, it is possible that this pathway could have been the cause of death in other neonatal MAS patients with early‐onset CS and cholestasis in which the cause of death was not conclusively established.^3^, ^24^, ^25^, ^26^, ^29^, ^30^, ^33^

The hepatobiliary dysfunction is another well‐established nonendocrine abnormality in MAS patients.^9^, ^10^ As is the case for *GNAS* mutation‐associated CS, the hepatobiliary dysfunction occurs when MAS appears early after birth.^3^, ^9^, ^10^, ^24^, ^25^, ^26^, ^27^, ^28^, ^29^, ^30^, ^31^, ^32^, ^33^, ^34^ It tends to progressively wane with age and has never been associated with a functional defect in the synthesis of hepatic factors or with intra‐ or extrahepatic bile ducts obstruction.^3^, ^9^, ^10^, ^25^, ^26^, ^30^, ^31^, ^32^, ^33^, ^34^ For example, in the two cases reported by Silva et al.,^26^ the last biopsies performed at 11 years and 9 years, respectively, revealed a “near normal” liver histology in both cases. Giant cell transformation of the hepatocytes, cholestasis, and a paucity of bile ducts have been the most common findings in liver histology.^3^, ^26^, ^31^ Recently, Coles and colleagues^34^ reported one patient who underwent liver transplantation at 10 months because of secondary complications including failure to thrive and recurrent infections. The histologic evaluation of the explanted liver revealed severe cholestatic disease with intrahepatic cholestasis, focal bile canalicular plugs, and mild‐to‐moderate focal periportal and sinusoidal fibrosis. In our case, giant cell transformation of hepatocytes was not observed. In contrast, an extensive portal‐based ductular reaction, the absence of biliary ducts within some portal tracts, intralobular biliary ducts at intrahepatic ectopic sites (away from the adjacent portal trat), bile plugs, and biliary metaplasia of the hepatocytes were the main pathological changes. Particularly prominent was the ductular reaction that reflected both the retrodifferentiation of hepatocytes to proliferating cholangiocytes and the activation of the hepatic progenitor cells at the interface between cholangioles and Hering's canals^51^, ^52^, ^53^ as highlighted, respectively, by the biliary metaplasia of hepatocytes and the evidence of CK7 positive cholangiocytes and CK7 negative hepatocytes in the lining of the newly generated ducts. Because cAMP is a well‐known inducer of cholangiocyte proliferation,^54^ the marked ductular reaction could be dependent on the intracellular increase of cAMP brought about by the mutation. Altogether these changes can be thought of as an effect of the *GNAS* mutation on the liver cells and may appear, as previously suggested,^3^ consistent with an imperfect morphogenesis of the intrahepatic bile duct system leading to cholestasis. As an additional finding, we also observed, by immunolocalization studies, an intracytoplasmic granular pattern of distribution of Gαs in the hepatocytes that contrasted with the predominant distribution of the protein along the sinusoidal domain of the hepatocytes of an age‐ and gender‐matched control. If and how the abnormal pattern of distribution of the Gαs contributes to the observed pathological changes deserves further in‐depth study.

In our case, as in other previously reported neonatal cases of MAS,^3^, ^23^, ^24^, ^25^, ^27^, ^28^, ^29^, ^30^, ^32^, ^33^, ^34^ hyperthyroidism and cardiac dysfunction were present as well. Although hyperthyroidism results from the presence of the Gαs mutation in thyroid tissue that leads to a ligand‐independent activation of the TSH/G‐protein/cAMP pathway with subsequent hyperplasia and hyperfunction of the gland,^3^ as well as an increase in thyroxine to triiodothyronine conversion,^55^ the pathogenesis of the cardiac dysfunction is more complex. It may manifest with cardiomegaly, ventricular hypertrophy, tachycardia, and sudden death. Even though the presence of cardiac cells harboring the mutation has been reasonably thought as responsible for both the cardiac dysfunction and the frequent premature and sudden death of MAS patients with early presentation,^3^ secondary responses to the increased metabolic demand induced by endocrine hyperfunction (hypercortisolism, hyperthyroidism) are also involved. Indeed, ventricular hypertrophy has been reported to decrease after medical therapy for CS and to resolve completely months after adrenalectomy^32^ and tachycardia, as in the case reported here, disappears with the pharmacological correction of the hyperthyroidism. Unfortunately, in our case, heart (and thyroid) samples were not available and neither mutational analysis nor histological evaluation could be performed.

We also observed additional postmortem findings including nephrocalcinosis and the presence of diffuse ductulo‐glandular complexes in the pancreas (ie, diffuse nesidioblastosis, congenital islet hyperplasia). Although the former has been previoulsy detected and can be related to the hypercortisolism,^3^, ^24^, ^25^, ^27^, ^28^, ^33^, ^56^, ^57^, ^58^ the latter has never been reported in neonatal MAS. In contrast, hypertrophic and hyperplastic islets were described in an adult MAS patient in the absence of hormonal hypersecretion.^15^ The *GNAS* gene is known to be expressed in the pancreas^59^ and *GNAS* mutations have been demonstrated in pancreatic samples obtained at autopsy from MAS patients^3^ and in pancreatic neoplasia occuring either within MAS or as an isolated disease.^11^, ^12^, ^13^, ^14^ We cannot establish definitely the pathogenetic mechanisms involved in the development of diffuse nesidioblastosis in our case. However, different mechanisms, not necessarily mutually exclusive, can be involved, including: (1) an exaggerated β‐cell response to the CS‐related hyperglycemia; (2) the effect of high levels of glucorticoids that may impair normal islet cell development^60^, ^61^; and/or (3) a direct contribution of the mutation, which, however, we could not demonstrate because of the unavailability of pancreatic tissue in this particular case.

In conclusion, the clinicopathologic and molecular study of this neonatal MAS patient, combined with a survey of previously reported cases, highlights a unique syndromic profile for neonatal MAS characterized by CAL skin macules, hypercortisolism, hyperthyroidism, cardiac and liver dysfunction, and the absence (or latency) of FD, and shows a frequent unfavorable outcome. This syndromic profile appears to be unique to the neonatal period. Because its clinical severity cannot be explained simply by the time of occurrence of the mutation,^16^ alternative hypotheses must be considered, such as, for example, the developmental fate of the specific region of the inner cell mass in which the mutation occurred. In particular, our case unequivocally proves that the different clinical expressions of neonatal MAS match closely the distribution of the disease genotype. Adrenal and liver disease dominated the clinical picture and harbored the highest numbers of mutated cells compared to other tissues/organs, including bone. The presence of low numbers of mutated cells in the other tissues and organs would have likely led to the subsequent development of lesions according to a specific temporal sequence, which, however, terminated prematurely because of the death of the patient. This could be a possible explanation for later onset of FD compared to nonskeletal abnormalities in the natural history of MAS.^62^ In addition, our case highlights how bone changes, unrelated to FD or to the direct impact of the disease genotype on bone, may contribute to the significant mortality of the patients with MAS presenting at neonatal age.

The knowledge and appreciation of the unusual clinical expressions of MAS in neonates may help clinicians to make a prompt diagnosis and implement immediate intervention.

## Disclosures

All the authors state that they have no conflicts of interest.
